# Patients referred to a German TMD-specialized consultation hour—a retrospective on patients without a diagnosis according to RDC/TMD decision trees

**DOI:** 10.1007/s00784-021-03866-z

**Published:** 2021-03-16

**Authors:** Angelika Rauch, Sebastian Hahnel, Anita Kloss-Brandstätter, Oliver Schierz

**Affiliations:** 1 Department of Prosthodontics and Materials Science, University of Leipzig, Liebigstr. 12, 04103 Leipzig, Germany; 2 Carinthia University of Applied Sciences, Europastr. 4, 9500 Villach, Austria

**Keywords:** Oral behaviors, Chronic pain, Craniomandibular disorders, Orofacial pain

## Abstract

**Objectives:**

The objective was to describe the physical and psychosocial features of patients attending a specialized consultation hour for temporomandibular disorders (TMD). This investigation focused on those patients who did not receive a diagnosis according to the Research Diagnostic Criteria for Temporomandibular Disorders (RDC/TMD).

**Materials and methods:**

From 2004 to 2017, patients seeking care during a TMD-specialized consultation hour were consecutively recruited. Each patient completed a TMD-related questionnaire, psychosocial questionnaires (Graded Chronic Pain Scale, Hospital Anxiety and Depression Scale, Beschwerden-Liste), and the Oral Health Impact Profile-49. The clinical examination was performed according to the RDC/TMD.

**Results:**

The mean age of the 1020 patients was 43.3 years (75.3% female). According to the RDC/TMD decision trees, 351 patients were categorized without a TMD diagnosis (*NoTMDdx*). The most frequent reasons for seeking care were orofacial pain/TMJ pain or headaches revealing an OR of 1.89 (for *TMDdx* group). A relevant proportion of patients was categorized as positive for anxiety (*NoTMDdx/TMDdx* 30.8/41.2%; *p* = 0.072), depression (20.8/23.9%; *p* = 0.433), non-specific physical symptoms (31.4/44.1%; *p* < 0.001), or dysfunctional chronic pain (11.5/18.2%; *p* < 0.001). In both patient groups, the oral health-related quality of life was impaired (42.9/52.7 points; *p* < 0.001), and the frequency of therapy measures prior to the consultation hour was high.

**Conclusions:**

Patients seeking care from TMD specialists were usually referred with TMD-associated symptoms. Of those, a relevant proportion did not receive a diagnosis according to RDC/TMD decision trees.

**Clinical relevance:**

Psychosocial screening and the avoidance of overtreatment are recommended for patients with TMD-related symptoms.

## Introduction

Patients suffering from non-dental pain or complaints in the orofacial area are a challenge in daily dental practice due to the complexity of the diagnostics. Within daily dental clinical routines, patients with orofacial pain are estimated to account for 19% of all patients [1]. When no dental-related reasons are obvious, general practitioners often refer these patients to specialists. The treatment cascade that follows basically involves ENT physicians, oral and maxillofacial surgeons and specialists on temporomandibular disorders (TMD) [1]. General practitioners estimate TMD to be the most frequent reason for orofacial pain symptoms [1, 2]. TMD has a complex etiopathology that is based on physical (axis I) and psychosocial (axis II) aspects. Therefore, a validated classification and diagnostic system is required that can be utilized for these patients in both research and clinical practice. The Research Diagnostic Criteria for Temporomandibular Disorders (RDC/TMD) are an internationally accepted system that fulfills these particular requirements by covering both physical examination and psychosocial assessment [3].

Previous studies have provided insight into TMD diagnoses according to RDC/TMD within patient clienteles. A systematic review by Manfredini et al. on axis I diagnoses concluded that the mean age of patients ranged from 30.2 to 39.4 years and the patient population included a high percentage of females [4]. The overall prevalence of muscle disorders was 45.3%, that of disc displacements (DD) was 41.1%, and that of joint disorders was 30.1%. These categories can be divided into subgroups in which myofascial pain without limited opening, DD with reduction, and arthralgia were the most frequent subdiagnoses. A recent Polish study on TMD patients observed similar frequencies of DD and joint disorders but higher frequencies of muscle disorders (56.9%) [5]. Regarding psychosocial characteristics, the investigation revealed that 10.5% of the patients presented with high pain-related impairment (grade III/IV on the Graded Chronic Pain Scale). Moreover, moderate-to-severe levels of somatization (non-specific physical symptoms) were observed in half of the patients, and moderate-to-severe levels of depression were observed in 37.6% of patients. The high prevalence values for psychosocial characteristics are well known in TMD patients [6].

Furthermore, dental patient-reported outcomes (dPRO) revealed that impairment of oral health-related quality of life (OHRQoL) in TMD patients is 2- to 3-fold higher than that in the general population [7]. The OHRQoL can be assessed with the Oral Health Impact Profile (OHIP) [8]. When introducing the OHIP in 1994, a 49-item version was made available, which has been reduced in terms of the number of items over the last few years. Today, the OHIP-49 and OHIP-14 are frequently used for research purposes.

Overall, specialized units or consultation hours for orofacial pain or TMD at hospitals/universities are visited frequently by patients seeking care [2]. The variety of TMD- and non-TMD-related complaints is supposed to be large, and a high diversity of psychosocial characteristics might be estimated.

The objective of this study was to describe the physical and psychosocial features of patients who attended a TMD-specialized consultation hour in a German university setting even if no TMD diagnosis according to RDC/TMD axis I decision trees was made. The focus was on the main complaints, psychosocial characteristics, and OHRQoL.

## Materials and methods

### Study design, setting, and participants

From 2004 to 2017, patients seeking care during a TMD-specialized consultation hour held at the Department of Prosthodontics and Materials Science at the University of Leipzig (Germany) were consecutively recruited. The consultation hour addressed patients who were referred by dentists from private practices or within the university. The appointment usually lasts for 60 min. In contrast to other areas of dentistry in Germany, the TMD examination is not covered by statutory health care but is covered by most private health insurances. The average waiting time ranged from 3 to 5 weeks. The examiners were experienced in TMD examination and treatment according to RDC/TMD guidelines but were not calibrated. Since 2004, the axis I and II results and demographics of German speaking patients have been collected in a database and data were retrospectively analyzed. All participants provided signed informed consent to contribute to this study. Ethical approval was obtained from the Ethics Committee of the University of Leipzig [146-2005]. For the analysis of the data, children and adolescents under the age of 18 years were excluded (excluded *n* = 37), as the RDC/TMD are only validated for adults [9].

### Psychosocial assessment and physical examination

The participants received various questionnaires 1 week before the initial appointment and were asked to complete them the day prior to the examination. During the consultation hour, the questionnaires were perused with the patients to clarify potential misunderstandings. The questionnaires gathered information on the perceived level of general and oral health, each of which was measured with a 5-point Likert scale (excellent, very good, good, moderate, poor). Furthermore, the German OHIP-49 was included. In addition, the main reason for attending the TMD-specialized consultation hour (only one answer possible) and the number of health care practitioners who had been visited due to the complaints were assessed in the questionnaires. Moreover, previous treatments related to the complaints were reported. For axis II assessment, the psychosocial questionnaires addressed the chronicity of pain within the last 6 months (Graded Chronic Pain Scale 1.0/GCPS) [10] as well as anxiety and depression (Hospital Anxiety Scale/HADS) [11]. A HADS cutoff value of ≥ 8 was used to define cases of anxiety or depression [12]. According to the manual of the RDC/TMD [13, 14], the Beschwerden-Liste (B-L) was used to assess non-specific physical symptoms; values ≥ 22 were used to categorize patient-reported impairments according to non-specific physical symptoms for both sexes [15].

During the consultation hour, all patients were examined according to the RDC/TMD. Over 13 years, the patients were examined by eight experienced dentists who were not calibrated. Moreover, no reliability values were assessed for the examiners. TMD diagnoses were computer-aided conducted with the help of digitalized RDC/TMD decision trees.

### Statistical analysis

Statistical analyses were performed with SPSS (IBM SPSS Statistics 27, IBM, Ehningen, Germany). For descriptive statistics of continuous variables, means and standard deviation were calculated; frequencies were determined for ordinal and categorical variables. The 95% CI of the prevalence values were constructed with bootstrapping assuming 1000 samples and a simple sampling approach. For continuous data, Cohen’s *d* was calculated. Mann-Whitney *U* tests were used for comparisons of ordinal and continuous data since the latter were not normally distributed (Shapiro-Wilk tests: *p* < 0.001). Regarding categorical variables, chi-square tests were performed for statistical comparisons. Dummy variables were created to compare the main reasons for seeking care between the two patient groups and odds ratios (OR) were calculated including 95% confidence intervals (95% CI). The level of significance was set to *p* < 0.050.

## Results

### Demographics, TMD-related patient history, and physical characteristics

**Fig. 1:**
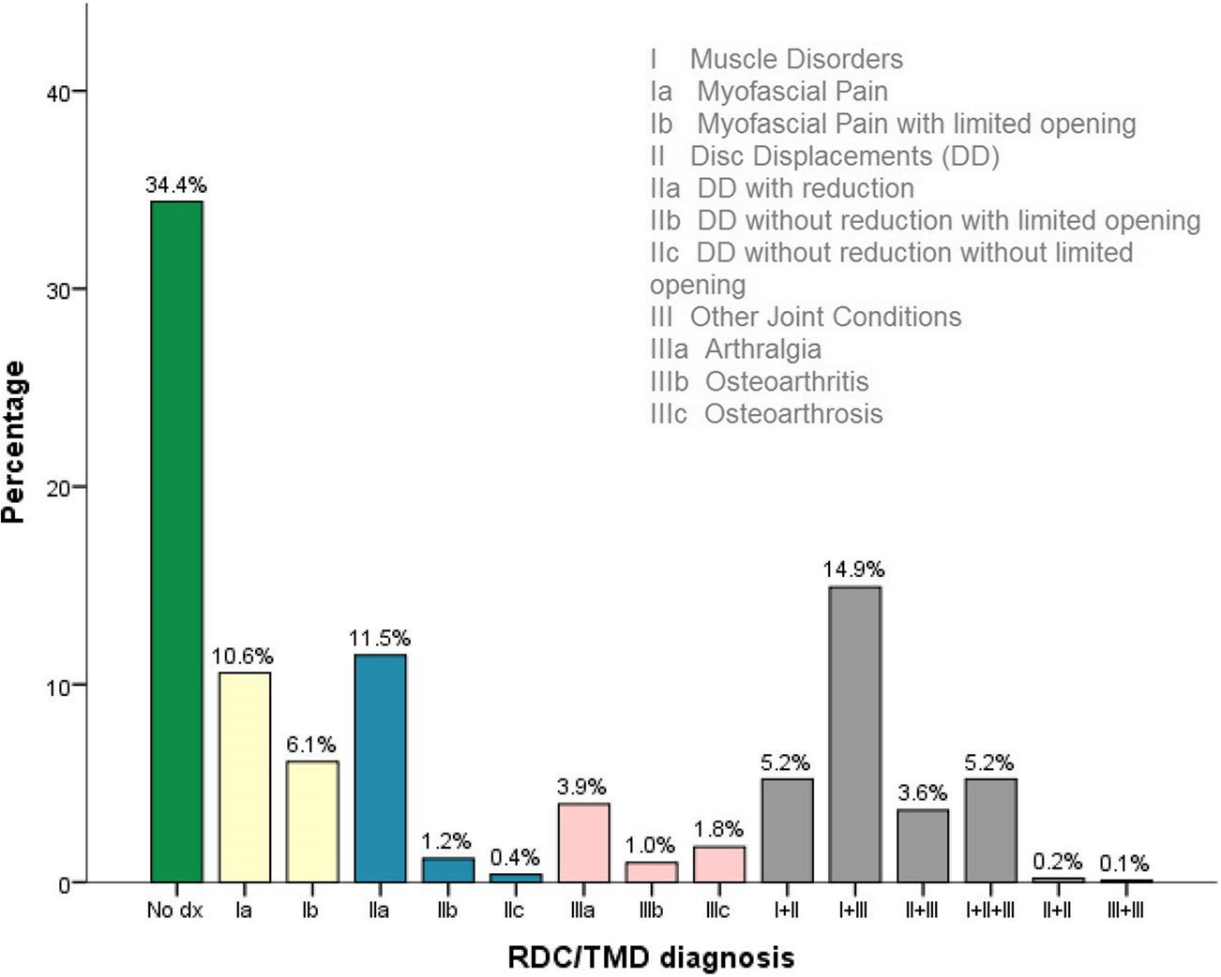
Frequency of RDC/TMD diagnoses in the patient population

After 13 years, data collection was completed for 1,020 patients. The mean age was 43.3 years (± 16.3 years; 18-83 years), and the patient population included 75.3% females. Eight dentists who were trained but had not received RDC/TMD calibration examined the patients. According to the RDC/TMD decision trees, 351 patients received no TMD diagnosis (34.4% [95% CI 31.6, 37.2]; *NoTMDdx group*), and 669 patients were given an RDC/TMD diagnosis (65.6% [95% CI 62.8, 68.4]; *TMDdx group*). Of those, 55.5% (*n* = 371) received a single diagnosis and 44.5% (*n* = 298) received multiple diagnoses (Fig. 1).

Upon comparing both groups, the age pattern was similar (*p* = 0.255), and women predominated in the gender distribution and comprised an even higher percentage of the *TMDdx* than the *NoTMDdx* group (68.7%/78.8%; *p* < 0.001). Within the *NoTMDdx* group, the main reasons for attending the specialized consultation hour were pain in the orofacial area/headache, limitations in jaw movements, and joint sounds. In the *TMDdx* group, the main reasons were “pain in the orofacial area/headache,” “joint sounds,” and a need for a “TMD examination” (Table 1). The differences in the main reasons for seeking care were statistically significant between both groups (*p* < 0.001). An OR of 1.89 [95% CI 1.44, 2.59] was calculated for the category “pain in the orofacial area/TMJ including headache” indicating a higher chance to be categorized as *TMDdx* group. Moreover, an OR of 0.56 [95% CI 0.37, 0.85] for “limitations of jaw movements,” an OR of 0.21 [95% CI 0.09, 0.51] for “bruxism/wear,” and an OR of 0.33 [95% CI 0.14, 0.77] for “tinnitus” were detected that suggest a lower chance to be categorized with an RDC/TMD diagnosis according to decision trees. Regarding other categories, all 95% CIs of the ORs included 1.00.

**Table 1:** Main reason for patients to seek care attending the TMD-specialized consultation hour according to patient’s answer (only one answer possible); *if applicable: no overlap of 95% confidence intervals (CI)

Category		*NoTMDdx* group (*N* = 351)		*TMDdx* group (*N* = 669)	*P* value
				Mean (SD)	
Age in years		44.0 (15.9)	-	43.0 (16.5)	0.255
	*Count*	Percentage [95% CI]	*Count*	Percentage [95% CI]	
Female	*241*	68.7% [63.2, 79.2]	*527*	78.8% [71.0, 80.1]	< 0.001
Main reasons for seeking care self-reported by patients (only one answer was possible)					* < 0.001
Pain in orofacial area/TMJ incl. headache	*106*	30.2% [25.9, 35.6]	*301*	44.9% [40.8, 49.2]	* < 0.001
Limitations of jaw movement	*46*	13.1% [9.7, 16.2]	*52*	7.8% [5.7, 9.9]	0.006
Joint sounds	*44*	12.3% [8.8, 15.4]	*73*	10.9% [8.5, 13.5]	0.522
TMD examination	*36*	0.3% [7.4, 13.4]	*65*	9.7% [7.3, 12.0]	0.784
Bruxism/wear	*17*	4.8% [2.6, 6.8]	*7*	1.1% [0.4, 1.9]	* < 0.001
Complaints in orofacial area/TMJ	*15*	4.3% [2.3, 6.5]	*20*	3.0% [1.9, 4.3]	0.285
Tinnitus	*14*	4.0% [2.2, 6.0]	*9*	1.4% [0.6, 2.2]	0.007
Others	*41*	11.9% [8.5, 15.7]	*65*	9.7% [7.8, 12.1]	-
No answer	*32*	9.1% [6.0, 12.3]	*77*	11.5% [9.1, 14.1]	-
Utilized therapy options prior to TMD consultation hour (N for *NoTMDdx/TMDdx* group*)*					
Prior splint therapy (130/318)	*70*	53.8% [45.4, 62.3]	*200*	62.9% [58.0, 68.5]	0.076
Prior physiotherapy (130/318)	*81*	62.3% [53.9, 70.0]	*198*	62.3% [57.1, 68.1]	0.993
Prior analgesics (121/309)	*26*	21.5% [16.2, 30.8]	*87*	28.2% [23.0, 33.1]	0.158
		Mean (SD)		Mean (SD)	
Prior health care practitioners (129/309)		3.2 (2.6)	-	3.5 (2.6)	0.171

Splint therapy, physiotherapy, and analgesics were used to a similar extent prior to the TMD-specialized consultation hour in both groups (*p* ≥ 0.076) (Table 1). Splints (*NoTMDdx/TMDdx* 53.8/62.9%) and previous physiotherapy (62.3/62.3%) were highly prevalent. In contrast, analgesics were chosen less often (21.5/28.2%). In general, patients had already consulted three health care practitioners (*p* = 0.171).

### Psychosocial characteristics

The evaluation of the axis II questionnaires revealed high proportions of patients with pain-related impairment (*NoTMDdx/TMDdx* 11.5/18.2%), anxiety (30.8/41.2%), depression (20.8/23.9%), and non-specific physical symptoms (31.4/44.1%). Statistically significant differences were observed for pain-related impairment and non-specific physical symptoms, which were more prevalent in the *TMDdx* group. The HADS-related outcomes for anxiety or depression were similar between the two groups (Table 2).

**Table 2:** Results of the psychosocial assessment, Oral Health Impact Profile (OHIP-49), and self-perceived health; if applicable: *count in italic*

Category	*NoTMDdx* group		*TMDdx* group		Difference [95% confidence interval of difference]	Cohen’s *d*	Significance P value
Mean (standard deviation)/percentage [95% CI] of axis II characteristics							
CPI (247/596)	*-*	4.3 (2.1)	*-*	5.3 (2.1)	− 1.0 [− 1.4, − 0.7]	0.50	< 0.001
DP (237/565)	*-*	0.7 (1.5)	*-*	1.1 (1.6)	-	-	0.029
GCPS (234/561)							
GCPS 0	*7*	3.0%	*4*	0.7%	-	-	< 0.001
GCPS I-II	*200*	86.5%	*455*	81.1%			
GCPS III-IV	*27*	11.5%	*102*	18.2%			
HADS (130/323)							
HADS sum score—anxiety	*-*	6.2 (4.0)	*-*	7.0 (4.4)	− 0.9 [− 1.7, 0.1]	0.20	0.072
HADS category—anxious	*40*	30.8% [23.3, 38.8]	*133*	41.2% [36.0, 46.7]	-	-	0.039
HADS sum score—depression	*-*	4.6 (3.9)	*-*	4.9 (4.0)	− 0.3 [− 1.1, 0.5]	0.08	0.433
HADS category—depressive	*27*	20.8% [14.0, 27.9]	*77*	23.9% [19.2, 28.7]	-	-	0.472
B-L (315/630)							
B-L sum score	*-*	16.8 (12.0)	*-*	20.3 (13.1)	− 3.5 [− 5.2, − 1.8]	0.41	< 0.001
B-L category - somatization	*99*	31.4% [26.0, 36.5]	*278*	44.1% [40.3, 47.8]	-	-	*< 0.001
Mean total and domain scores (standard deviation) of the OHIP-49							
Total score (312/611)	42.9 (31.2)		52.7 (33.1)		− 9.8 [− 14.2, − 5.5]	0.30	< 0.001
Functional limitation	-	8.1 (5.9)	-	9.5 (5.9)	− 1.4 [− 2.2, − 0.6]		< 0.001
Physical pain		9.9 (7.3)		12.6 (7.6)	− 2.7 [− 3.7, − 1.7]		< 0.001
Psychological discomfort		6.4 (5.0)		7.2 (5.0)	− 0.8 [− 1.5, − 0.1]		0.013
Physical disability		5.2 (5.7)		7.1 (6.6)	− 1.9 [− 2.7, − 1.0]		< 0.001
Psychological disability		6.0 (5.3)		7.2 (5.5)	− 1.2 [− 1.9, − 0.5]		0.001
Social disability		2.8 (3.4)		3.6 (3.9)	− 0.8 [− 1.3, − 0.3]		0.003
Handicap		4.6 (4.7)		5.6 (5.1)	− 1.1 [− 1.7, − 0.4]		0.002
Percentage of answers for self-perceived health							
General health (307/605)							
Excellent	*21*	6.8%	*44*	7.3%	-	-	0.001
Very good	*58*	18.9%	*183*	30.2%			
Good	*158*	51.5%	*279*	46.1%			
Moderate	*60*	19.5%	*80*	13.2%			
Poor	*10*	3.3%	*19*	3.1%			
Oral health (304/595)							
Excellent	*46*	15.1%	*95*	16.0%	-	-	0.518
Very good	*101*	33.2%	*213*	35.8%			
Good	*128*	42.1%	*220*	37.0%			
Moderate	*25*	8.2%	*59*	9.9%			
Poor	*4*	1.3%	*8*	1.3%			

*CPI* chronic pain intensity, *DP* disability points, *GCPS* Graded Chronic Pain Scale, *HADS* Hospital Anxiety Scale, *B-L* Beschwerden-Liste

### Oral health-related quality of life

High OHIP sum scores were identified, yet sum scores were significantly increased for *TMDdx* patients (*p* < 0.001) (Table 2). However, significantly more patients in the *TMDdx* group rated their perceived general health as more positive than those in the *NoTMDdx* group (*p* = 0.001), although perceived oral health was broadly similar in both groups (*p* = 0.518).

## Discussion

The results of the present study revealed that patients who did not receive any axis I diagnosis according to RDC/TMD decision trees were seeking care from TMD specialists with similar main complaints as those of patients who received a diagnosis. The main complaints were described as pain in the orofacial area, limitations in jaw movements, and joint sounds. According to psychosocial assessment, 10.2% of the *NoTMDdx* group described pain-related impairments, and 20–30% had positive findings for anxiety, depression, or non-specific physical symptoms. Every tenth patient perceived pain-related impairments. Moreover, the oral health-related quality of life was impaired. Nonetheless, the scoring results for anxiety, non-specific symptoms, pain-related impairments, and OHRQoL revealed lower values in the *NoTMDdx* than in the *TMDdx* group.

The age pattern and proportion of women in both patient groups were comparable to the results of a systematic review on TMD patients [4]. The latter presented similar prevalence values for subdiagnoses compared to those in the *TMDdx* group. Myofascial pain and DD with reduction were highly pronounced. In contrast, arthralgia was less common in the recent patient population than in the population examined in the systematic review. The number of patients who did not meet the criteria for any RDC/TMD diagnosis (34.4%) was high in comparison to the results for an Italian patient population (16.4%) [16]. This phenomenon might result from the peculiarities of the German health care system, as the number of TMD-specialized units is relatively low in comparison to that in other countries.

In Germany, specialization in the area of TMD is an extra qualification, and qualified dentists usually continue to work in prosthodontics, orthodontics, or maxillofacial medicine. Thus, it can be assumed that there are fewer possibilities to attend appointments with TMD specialists in Germany than in other countries. Moreover, recent surveys on non-dental orofacial pain/TMD diagnoses and treatment in Sweden and Germany revealed that general dentists desire an improvement in undergraduate/postcurricular TMD/orofacial pain education [1, 17, 18]. This observation might serve as an explanation for the diversity and the high number of patients referred to German TMD specialists. The uncertainty could also result in the referral of patients with easier-to-treat symptoms, such as difficulties with mandibular movements or joint sounds. Nonetheless, these symptoms cause patients to seek care, since they are afraid to injure themselves [19]. Consequently, they might “urge” dentists to present any treatment option. On the one hand, demand for treatment might explain the high prescription rates of splints and physiotherapy for *NoTMDdx* patients in the present study. These treatment options are financially covered by German statutory health care, while diagnosis is only covered by most private health insurances. However, the authors assume the percentage of TMD patients with private health insurance who attend the specialized consultation hour to be less than 10%. On the other hand, the demand for treatment might be a reason for referral to TMD specialists. Consequently, continuous education of colleagues and patients could decrease the variety and number of visited health care practitioners, since, for instance, pain-free TMJ clicking usually does not require any intervention.

The classification of the *TMDdx* group according to RDC/TMD decision trees might be a reason for the high values of *NoTMDdx* patients, as the decision trees are only supposed to reveal the core of TMD diagnoses. Besides, painful TMD conditions are well represented in the decision trees, which are corroborated by the results of the present study revealing an OR of 1.89 for painful main complaints, whereas, e.g., limitations of jaw movements had an OR of 0.56. Thus, an expanded taxonomy of TMD diagnoses [20] or other taxonomies, e.g., that of the American Association of Orofacial Pain, could have been applied for *NoTMDdx* patients. As the collection of the presented data had already begun in 2004, the only available two-axis diagnostic system in the German language was the RDC/TMD, and only diagnoses established based on the RDC/TMD decision trees were gathered in the database. Since the end of 2018, the translation of the Diagnostic Criteria for Temporomandibular Disorders (DC/TMD) into German has been approved by the international INFoRM consortium [21]. The DC/TMD introduced revised axis I decision trees that include a subgroup for “headache attributed to TMD.” Thus, the application of the DC/TMD might decrease the number of *NoTMDdx* patients since headache was one of the main complaints in the present study.

Moreover, some symptoms reported by the patients in the current study are considered controversial. In particular, the presence of tinnitus is reported by a vast number of TMD patients and might be regarded as a comorbidity, but evidence from previous investigations is low [22, 23]. Other main complaints, especially in the *NoTMDdx* group, that were summarized as “others” in Table 1 were described as follows: uncomfortable bite, pain in parts of the body other than the orofacial area, vertigo, burning mouth, or problems with swallowing. Some of these symptoms, e.g., problems with swallowing, might be affected by TMD, but the correlation has been only marginally investigated [24].

Psychosocial characteristics may be a relevant factor for a high number of patients without any TMD diagnosis. A total of 20–30% of the *NoTMDdx* patients had positive results for anxiety, depression, or non-specific physical symptoms. Every tenth patient perceived pain-related impairments. These values are striking and should indicate that dentists and specialists should keep psychosocial modulators in mind. Thus, it seems important to address possible psychosocial origins early and to clarify psychosocial modulators with a multidisciplinary approach, where applicable.

During the 13-year period, a total of eight dentists examined the patients during a TMD-specialized consultation hour, but none of them was calibrated, which can be seen as a limitation of this study. However, a current investigation focusing on DC/TMD diagnosis revealed that self-instruction can be a reliable approach to teach examiners [25]. Another limiting point was that all of the patients referred to the TMD-specialized consultation hour presented a self-perceived treatment need, but the actual treatment was not recorded in the database. In addition, the knowledge of dentists and access to TMD specialist/units or first-line treatments is hard to generalize, as heterogeneity is high worldwide. This is mostly due to variations in undergraduate/postgraduate education and the financial coverage of health care. However, this investigation presents data from a large population of German patients who were seeking care due to TMD-associated symptoms while applying a validated and internationally established diagnostic system. The demographics of the German patient population were similar to results from other countries. Moreover, the present study provides insight into the perceived general and oral health and the oral health-related quality of life of these patients.

The data of the present study might help to adjust current research focusing on complaints that are not covered by the diagnostic algorithms, revise therapeutic interventions by dentists, and accompany ongoing modifications of the taxonomies of temporomandibular disorders or orofacial pain-related diagnostic criteria.
